# Increased Risk of Psychiatric Disorders in Allergic Diseases: A Nationwide, Population-Based, Cohort Study

**DOI:** 10.3389/fpsyt.2018.00133

**Published:** 2018-04-24

**Authors:** Nian-Sheng Tzeng, Hsin-An Chang, Chi-Hsiang Chung, Yu-Chen Kao, Chuan-Chia Chang, Hui-Wen Yeh, Wei-Shan Chiang, Yu-Ching Chou, Shan-Yueh Chang, Wu-Chien Chien

**Affiliations:** ^1^Department of Psychiatry, Tri-Service General Hospital, School of Medicine, National Defense Medical Center, Taipei, Taiwan; ^2^Student Counseling Center, National Defense Medical Center, Taipei, Taiwan; ^3^Department of Medical Research, Tri-Service General Hospital, National Defense Medical Center, Taipei, Taiwan; ^4^Taiwanese Injury Prevention and Safety Promotion Association, Taipei, Taiwan; ^5^School of Public Health, National Defense Medical Center, Taipei, Taiwan; ^6^Department of Psychiatry, Tri-Service General Hospital, Song-Shan Branch, National Defense Medical Center, Taipei, Taiwan; ^7^Institute of Bioinformatics and Systems Biology, National Chiao Tung University, Hsin-Chu, Taiwan; ^8^Department of Nursing, Tri-Service General Hospital, School of Nursing, National Defense Medical Center, Taipei, Taiwan; ^9^Department of Nursing, Kang-Ning University (Taipei Campus), Taipei, Taiwan; ^10^Division of Chest and Critical Medicine, Department of Medicine, Tri-Service General Hospital, School of Medicine, National Defense Medical Center, Taipei, Taiwan; ^11^Graduate Institute of Medical Sciences, National Defense Medical Center, Taipei, Taiwan; ^12^Graduate Institute of Life Sciences, National Defense Medical Center, Taipei, Taiwan

**Keywords:** bronchial asthma, allergic rhinitis, atopic dermatitis, psychiatric disorders, risk factors, cohort study, Taiwan National Health Insurance Program, National Health Insurance Research Database

## Abstract

**Background/objective:**

Allergic diseases, such as bronchial asthma, allergic rhinitis, atopic dermatitis, and psychiatric disorders, are major health issues. There have been reports that allergic diseases were associated with depression or anxiety disorders. This study aimed to investigate the association between these allergic diseases and the risk of developing overall psychiatric disorders in patients from Taiwan.

**Methods:**

This cohort study used the database of the Taiwan National Health Insurance Program. A total of 186,588 enrolled patients, with 46,647 study subjects who had suffered from allergic diseases, and 139,941 controls matched for sex and age, from the Longitudinal Health Insurance Dataset of 2000–2015, were selected from a sub-dataset of the National Health Insurance Research Database. Fine and Gray’s competing risk model analysis was used to explore the hazard ratio (HR), and 95% confidence interval, for the risk of allergic diseases being associated with the risk of developing psychiatric disorders during the 15 years of follow-up.

**Results:**

Of the study subjects, 5,038 (10.8%) developed psychiatric disorders when compared to 9,376 (6.7%) in the control group, with significant difference (*p* < 0.001). Fine and Gray’s competing risk model analysis revealed that the adjusted HR was 1.659 (95% CI = 1.602–1.717, *p* < 0.001). In this study, we found that the groups of atopic dermatitis alone and the allergic rhinitis + atopic dermatitis were associated with a lower risk of psychiatric disorders, but all the other four groups, such as bronchial asthma alone, allergic rhinitis alone, bronchial asthma + allergic rhinitis, bronchial asthma + atopic dermatitis, and the combination of all these three allergic diseases, were associated with a higher risk of psychiatric disorders.

**Conclusion:**

Allergic diseases are therefore associated with a 1.66-fold increased hazard of psychiatric disorders in Taiwan.

## Introduction

Child and adolescent allergic diseases, such as bronchial asthma, allergic rhinitis, and atopic dermatitis, are common and have made an impact on the patients’ physical health (1–3). Furthermore, previous studies have shown the association between asthma and anxiety (4), depressive, bipolar, and overall mood disorders (5, 6), attention-deficit hyperactivity disorder (ADHD) (7, 8), schizophrenia (9), and even dementia (10). Some studies have also demonstrated the associations between allergic rhinitis and depressive disorder (11, 12) or bipolar disorder (13). The association between atopic dermatitis and depressive and anxiety disorders has also been reported (14). However, another study found that allergic diseases such as rhinitis or urticarial are associated with a lower risk of schizophrenia (15). Therefore, a study is needed for the overall consideration of the association between these three common allergic diseases and important psychiatric disorders.

Psychiatric, or mental, disorders are defined as clinically significant behavioral or psychological syndromes, which are associated with present distress, disability, or an increased risk of suffering death, pain, or disability, and subsequent behavioral, psychological, or biological dysfunctions (16, 17). Previous studies have found that some psychiatric disorders are associated with several inflammatory diseases (18, 19), such as multiple sclerosis (20, 21), fibromyalgia (22, 23), or inflammatory bowel diseases (24). Psychological stressors related to these diseases might also contribute to both psychiatric and physical morbidity (25, 26).

Recent studies have been increasing on the interactions between allergy-related inflammatory or immunological factors and psychiatric disorders, such as depression (27), anxiety (28), bipolar disorder, and schizophrenia (29). However, further study is still needed to clarify the mechanisms underlying the association between allergic diseases and psychiatric disorders. Nevertheless, the association between allergic diseases and psychiatric disorders has not, as yet, been studied. Therefore, a nationwide, population-based study is necessary for the association between allergic diseases and the risk of psychiatric disorders for the clinicians who actually care for these patients.

## Materials and Methods

### Data Sources

The National Health Insurance (NHI) Program was launched in Taiwan in 1995, and as of June 2009, included contracts with 97% of the medical providers with approximately 23 million beneficiaries or more than 99% of the entire population (30, 31). The National Health Insurance Research Database (NHIRD), which contains all the claims data of the beneficiaries, uses the International Classification of Diseases, 9th Revision, Clinical Modification (ICD-9-CM) codes to record diagnoses (32). The details of the program have been documented in previous studies (22, 33–42).

A subset of the NHIRD, Longitudinal Health Insurance Database of a two million randomized sampled population in 2000–2015, was used to study the association between allergic diseases and the risk of psychiatric disorders. The present study used the NHIRD to identify patients with the diagnosis of allergic diseases, based on the ICD-9-CM codes, such as bronchial asthma (ICD-9-CM: 493.x), allergic rhinitis (ICD-9 CM code 477.X), and atopic dermatitis (ICD-9-CM code 691.X) during this period. The Institutional Review Board of the Tri-Service General Hospital approved this study and waived the need for individual written informed consent (IRB No. 2-105-05-040 and IRB No. 2-105-05-082).

### Study Design and Sampled Participants

**Figure 1:**
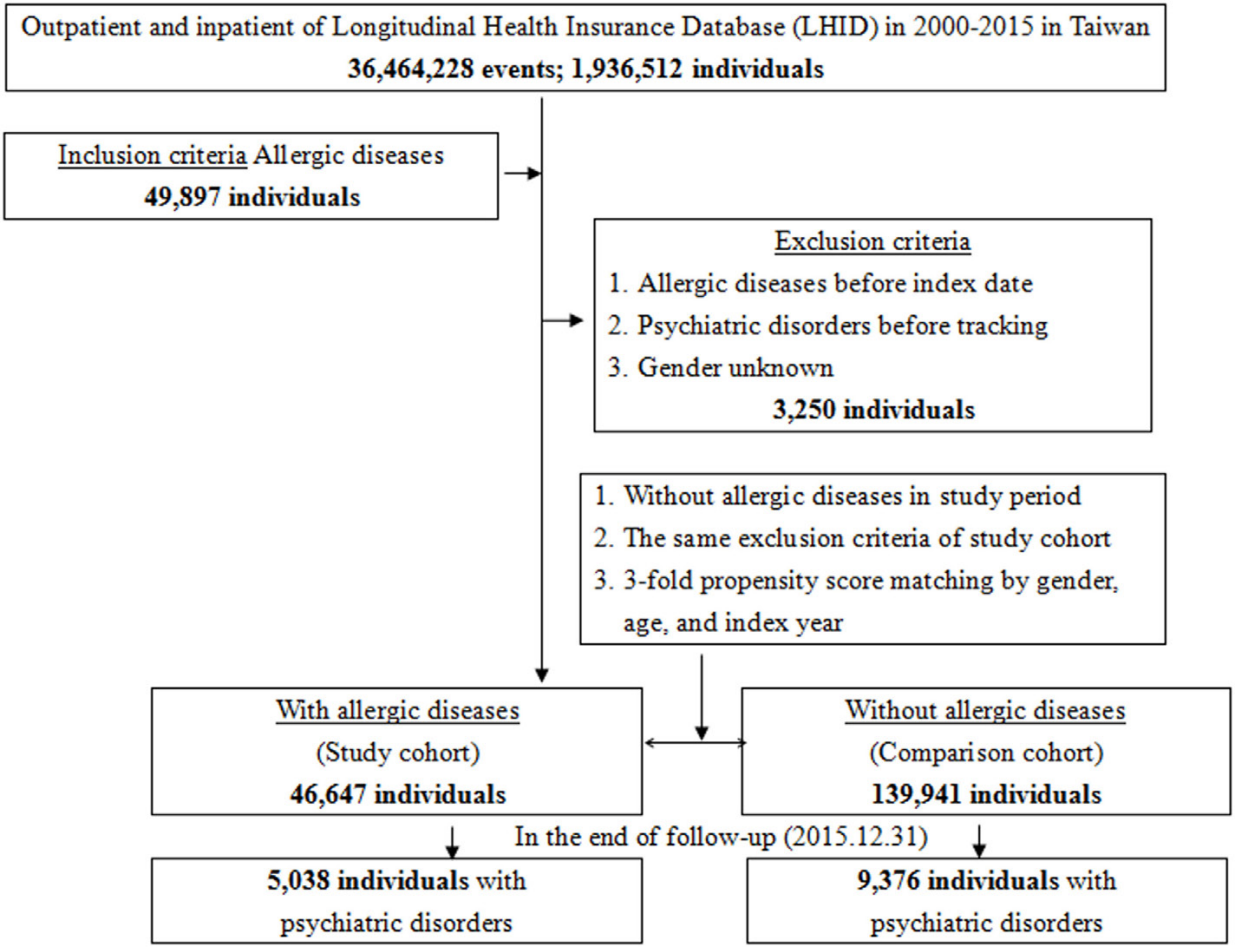
Flowchart of the study sample selection from the National Health Insurance Research Database in Taiwan.

This study was of a population-based, matched-cohort design. Patients with newly diagnosed bronchial asthma, allergic rhinitis, or atopic dermatitis were selected from the Longitudinal Health Insurance Database from January 1, 2000, to December 31, 2015. The patients with asthma, allergic rhinitis, or atopic dermatitis before 2000 were excluded. This method could be viewed as a wash-out period to make sure all the allergic diseases were recent onset with references from other studies for the association between allergic diseases and psychiatric morbidity, using the NHIRD (6, 7, 11, 15). This wash out period method could avoid the carry-over bias from exposure to the pre-existing allergic diseases. In addition, the patients diagnosed with dementia, depressive disorders, anxiety disorders, eating disorders, bipolar disorders, sleep disorders, and psychotic disorders, before 2000, or before their first visit for asthma, allergic rhinitis, or atopic dermatitis were also excluded. Each enrolled patient was required to have made at least three outpatient visits within the 1-year study period for allergic diseases according to these ICD-9-CM codes. A total of the patients who were enrolled, including 46,647 subjects with allergic diseases and 139,941 controls without allergic diseases, were matched for age, sex, and index date (Figure 1).

### Covariates

The covariates included sex, age groups (<20, 20–49, ≥50 years), geographical area of residence (north, center, south, and east of Taiwan), urbanization level of residence (levels 1–4), and monthly income (in New Taiwan Dollars; <18,000, 18,000–34,999, and ≥35,000). The urbanization level of residence was defined according to the population and various indicators of the level of development. Level 1 was defined as a population of >1,250,000, and a specific designation as political, economic, cultural, and metropolitan development. Level 2 was defined as a population between 500,000 and 1,249,999 and as playing an important role in the politics, economy, and culture. Urbanization levels 3 and 4 were defined as a population between 149,999 and 499,999, and <149,999, respectively (43).

### Comorbidities

Comorbidities were assessed using the Charlson Comorbidity Index (CCI), which categorizes the comorbidities using the ICD-9-CM codes, scores each comorbidity category (44–49), and combines all scores to calculate a single comorbidity score. A score of zero indicates that no comorbidities were found, and higher scores indicate higher comorbidity burdens (31).

### Major Outcome

All of the study participants were followed from the index date until the onset of dementia (ICD-9-CM codes: 290.0, 290.10, 290.11, 290.12, 290.13, 290.20, 290.21, 290.3, 290.41, 290.42, 290.43, 290.8, 290.9, and 331.0), anxiety disorders (ICD-9-CM 300), depressive disorders (ICD-9-CM 296.2–296.3, 300.4, and 311), eating disorders (anorexia nervosa 307.1, bulimia nervosa 307.51, and other disorders of eating 307.59), bipolar disorders (ICD-9-CM 296.0, and 296.4–296.8), sleep disorders (ICD-9-CM 307.4 and 780.5), and psychotic disorders (ICD-9-CM 295 and 297–298), withdrawal from the NHI program, or the end of 2015. In addition, each psychiatric diagnosis was required to have made at least three outpatient visits within the 1-year study period for psychiatric disorders according to these ICD-9-CM codes.

### Statistical Analyses

All statistical analyses were performed using the SPSS for Windows, version 22.0 (IBM Corp., Armonk, NY, USA). χ^2^ and *t* tests were used to evaluate the distributions of the categorical and continuous variables, respectively, with a Fisher’s exact examination. Fine and Gray’s competing risk analysis was used to determine the risk of psychiatric disorders since death can act as a competing risk factor for psychiatric disorders (40, 50, 51). The results were presented as hazard ratio (HR) with a 95% confidence interval (CI). Differences in the risk of psychiatric disorders between the study and control groups were estimated using the Kaplan–Meier method with the log-rank test. A two-tailed *p* value < 0.05 was considered to indicate the statistical significance.

## Results

### Baseline Characteristics of the Study Population

The baseline characteristics of the study population are depicted in Table 1. There were 46,647 subjects in the allergic diseases group and 139,941 non-allergic diseases in the control group, with a similar distribution of sex, age, marital status, education years, and monthly insured premiums. The mean CCR (SD) for the subjects was 1.32 (1.10) and 0.67 (1.68) for the allergic diseases and control group, respectively. The subjects had more medical visits in the spring and winter, with residence in the south, east, and offshore islands of Taiwan, living in levels 3 and 4 in urbanization, or seeking help in local hospitals than the control group (*p* < 0.001 for all). In the subjects with allergic diseases, 40,405 have asthma, 1,809, allergic rhinitis, and 4,433, atopic dermatitis.

**Table 1:** Characteristics of study at the baseline.

Allergic diseases	With		Without		
	
Variables	*n*	%	*n*	%	*p*
**Total**	46,647	25.00	139,941	75.00	
**Gender**					0.999
Male	27,162	58.23	81,486	58.23	
Female	19,485	41.77	58,455	41.77	
**Age (years)**	44.82 ± 30.58		44.83 ± 27.77		0.948
**Age group (years)**					0.999
<20	14,766	31.65	44,298	31.65	
20–49	6,174	13.24	18,522	13.24	
≥50	25,707	55.11	77,121	55.11	
**Married**					0.147
Without	20,526	44.00	61,476	43.62	
With	26,121	56.00	79,465	56.38	
**Education (years)**					0.421
<12	13,757	29.49	40,997	29.30	
≥12	32,890	70.51	98,944	70.70	
**Insured premium (New Taiwan Dollars)**					0.437
<18,000	37,275	79.91	111,495	79.67	
18,000–34,999	5,520	11.83	16,642	11.89	
≥35,000	3,852	8.26	11,804	8.43	
**CCI_R**	1.32 ± 1.10		0.67 ± 1.68		<0.001
**Season**					<0.001
Spring (March–May)	12,770	27.38	37,326	26.67	
Summer (June–August)	11,376	24.39	35,204	25.16	
Autumn (September–November)	10,095	21.64	30,807	22.01	
Winter (December–February)	12,406	26.60	36,604	26.16	
**Location**					<0.001
Northern Taiwan	16,460	35.29	56,914	40.67	
Middle Taiwan	12,150	26.05	38,437	27.47	
Southern Taiwan	13,324	28.56	35,541	25.40	
Eastern Taiwan	4,398	9.43	8,117	5.80	
Outlets islands	315	0.68	932	0.67	
**Urbanization level**					<0.001
1 (the highest)	12,504	26.81	50,018	35.74	
2	18,131	38.87	59,219	42.32	
3	4,319	9.26	9,957	7.12	
4 (the lowest)	11,693	25.07	20,747	14.83	
**Level of care**					<0.001
Hospital center	8,046	17.25	45,001	32.16	
Regional hospital	13,379	28.68	43,677	31.21	
Local hospital	25,222	54.07	51,263	36.63	

*p: Chi-square/Fisher’s exact test on category variables and t-test on continue variables*.

*Without married: un-married, divorce, spouse death, and unknown*.

*CCI_R, Charlson comorbidity index removed dementia*.

### Allergic Diseases Associated With Psychiatric Disorders

**Figure 2:**
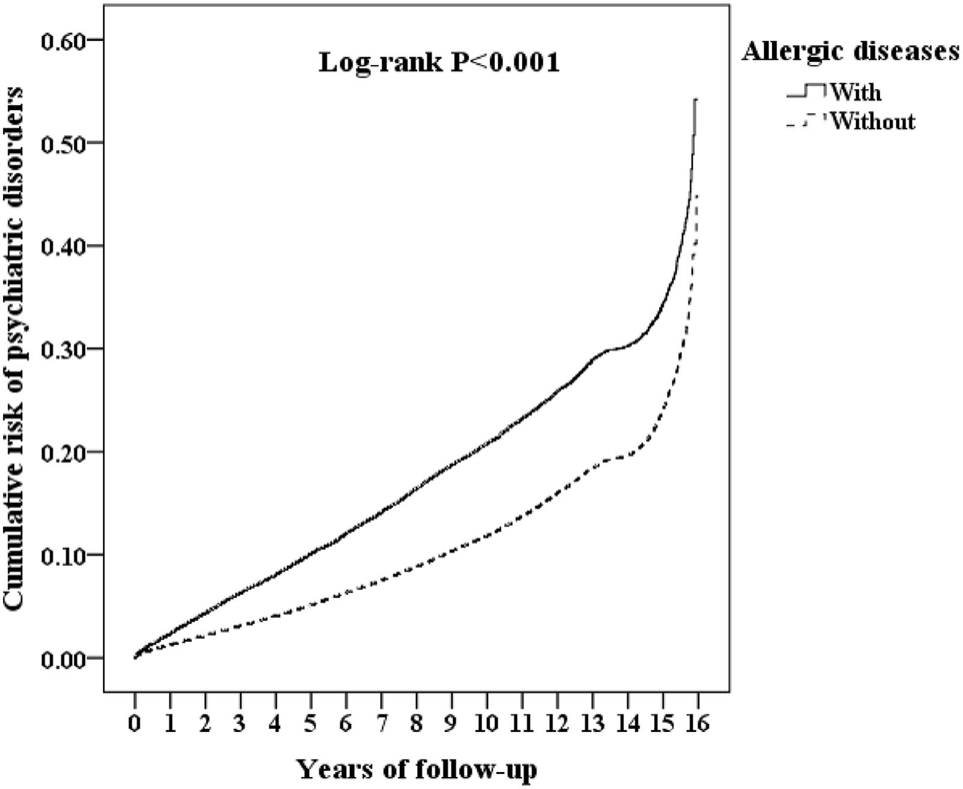
Kaplan–Meier for the cumulative risk of psychiatric disorders stratified by allergic diseases with the log-rank test.

Of the study subjects, 5,038 (10.8%) developed psychiatric disorders when compared to 9,376 (6.7%) in the control group, in the 15-year follow-up. We have also examined the risk of psychiatric disorders associated with allergic diseases. After adjusting for age, sex, CCI scores, and all the covariates, the Fine and Gray’s survival analysis revealed that the adjusted HR for psychiatric disorders was 1.659 for the subjects (95% CI = 1.602–1.717, *p* < 0.001) when compared with the control group. With reference to the female subjects, the male subjects were associated with a higher risk of psychiatric disorders. With reference to the younger group (aged < 20 years), older age groups (aged 20–49 years and aged ≥ 50 years) were associated with a higher risk of psychiatric disorders (*p* < 0.001) (Table 2). Figure 2 shows the Kaplan–Meier analysis for the cumulative incidence of psychiatric disorders in the study subjects and the control groups (log-rank test, *p* < 0.001).

**Table 2:** Factors of psychiatric disorders by using Fine and Gray’s competing risk model.

Model	Competing risk in the model			

Variables	Adjusted HR	95% CI	95% CI	*p*
**Allergic diseases**				
Without	Reference			
With	1.659	1.602	1.717	<0.001
**Gender**				
Male	1.108	1.072	1.146	<0.001
Female	Reference			
**Age group (years)**				
<20	Reference			
20–49	3.807	3.375	4.293	<0.001
≥50	8.747	7.819	9.784	<0.001

*HR, hazard ratio; CI, confidence interval; adjusted HR, adjusted variables listed in Table 1*.

Furthermore, each single increased score of CCI was associated with a 2% increased risk of psychiatric disorders, and study subjects who sought medical help in the summer (adjusted HR 0.914, *p* < 0.001), autumn (adjusted HR 0.748, *p* < 0.001), and winter (adjusted HR 0.921, *p* < 0.001) were associated with a lower risk of psychiatric disorders (data not shown). Study subjects who live in urbanization level 3 were associated with a lower risk of psychiatric disorders than those in level 4 (adjusted HR 0.817, *p* < 0.001). Study subjects who sought medical help in the medical centers (adjusted HR 0.504, *p* < 0.001) and regional hospitals (adjusted HR 0.635, *p* < 0.001) were associated with a lower risk of psychiatric disorders than those in the local hospitals (data not shown).

### Association Between Different Allergic Diseases and Risk of Psychiatric Disorders

Table 3 reveals that the association between each group of allergic diseases and the individual psychiatric disorders using Fine and Gray’s competing risk model. The subjects with atopic dermatitis were associated with a decreased risk of overall and individual psychiatric disorders.

**Table 3:** Factors of psychiatric disorders subgroup stratified by allergic diseases subgroup by using Fine and Gray’s competing risk model.

Allergic diseases (with vs. without)				

Psychiatric disorders subgroup	Adjusted HR	95% CI	95% CI	*p*
Overall	1.659	1.602	1.717	<0.001
Dementia	1.193	1.152	1.234	<0.001
Anxiety	2.397	2.315	2.481	0.009
Eating disorders	2.646	2.555	2.739	<0.001
Anorexia nervosa	2.573	2.485	2.663	0.003
Bulimia nervosa	–	–	–	–
Other disorders of eating	3.087	2.981	3.195	0.003
Depression	1.412	1.364	1.461	<0.001
Bipolar disorders	1.124	1.086	1.163	0.001
Sleep disorders	2.302	2.223	2.383	0.007
Psychotic disorders	1.192	1.151	1.234	0.003

Asthma	1.780	1.719	1.842	<0.001
Dementia	1.293	1.249	1.339	0.007
Anxiety	2.579	2.491	2.669	<0.001
Eating disorders	2.943	2.842	3.046	0.010
Anorexia nervosa	2.861	2.763	2.961	0.003
Bulimia nervosa	–	–	–	–
Other disorders of eating	3.434	3.316	3.554	<0.001
Depression	1.482	1.431	1.533	0.004
Bipolar disorders	1.180	1.139	1.221	<0.001
Sleep disorders	2.483	2.398	2.570	<0.001
Psychotic disorders	1.260	1.217	1.304	0.001

Allergic rhinitis	1.156	1.117	1.197	<0.001
Dementia	0.329	0.317	0.340	0.027
Anxiety	1.806	1.744	1.870	0.001
Eating disorders	0.000	–	–	0.989
Anorexia nervosa	0.000	–	–	0.976
Bulimia nervosa	–	–	–	–
Other disorders of eating	0.000	–	–	0.992
Depression	1.818	1.756	1.882	<0.001
Bipolar disorders	1.303	1.258	1.349	<0.001
Sleep disorders	1.342	1.296	1.389	<0.001
Psychotic disorders	1.331	1.285	1.377	<0.001

Allergic dermatitis	0.256	0.247	0.265	0.031
Dementia	0.272	0.263	0.282	0.030
Anxiety	0.190	0.183	0.196	0.025
Eating disorders	0.000	–	–	0.986
Anorexia nervosa	0.000	–	–	0.977
Bulimia nervosa	–	–	–	–
Other disorders of eating	0.000	–	–	0.993
Depression	0.211	0.204	0.219	0.028
Bipolar disorders	0.246	0.238	0.255	0.034
Sleep disorders	0.317	0.306	0.328	0.006
Psychotic disorders	0.162	0.156	0.167	0.005

*PYs, person-years; adjusted HR, adjusted hazard ratio: adjusted for the variables listed in Table 1; CI, confidence interval*.

In addition, the patients with allergic diseases who live in the middle (adjusted HR: 1.118, *p* < 0.001), southern (adjusted HR: 1.055, *p* = 0.003), and eastern Taiwan (adjusted HR: 1.596, *p* < 0.001) showed slightly higher risk of psychiatric disorders, with reference of those who live in northern Taiwan. The patients who live in area of urbanization level 3 (adjusted HR: 0.763, *p* < 0.001) showed lower risk of psychiatric disorders, with reference of those who live in the urbanization level 4. The patients who sought for medical help in the hospital centers (adjusted HR: 0.504, *p* < 0.001) and regional hospitals (adjusted HR: 0.635, *p* < 0.001) showed lower risk of psychiatric disorders with the reference of local hospitals (Data not shown).

Table 4 reveals the adjusted HR of affective disorders (adjusted HR: 1.385, *p* = 0.001, in which depression with adjusted HR: 1.412, *p* = 0.001, and bipolar disorders with adjusted HR: 1.124, *p* = 0.001), anxiety disorders (adjusted HR: 2.397, *p* = 0.009), psychotic disorders (adjusted HR: 1.192 *p* = 0.003), and dementia (adjusted HR: 1.193, *p* < 0.001) in patients with allergic diseases, when compared to the patients those without allergic diseases.

**Table 4:** Hazard ratio (HR) of affective, anxiety, psychotic disorders, and dementia in patients with vs. without allergic diseases.

Allergic diseases (with vs. without)	Competing risk in the model			

Psychiatric disorders subgroup	Adjusted HR	95% confidence interval (CI)	95% CI	*p*
Affective disorders	1.385	1.337	1.434	0.001
Depression	1.412	1.364	1.461	<0.001
Bipolar disorders	1.124	1.086	1.163	0.001
Anxiety disorders	2.397	2.315	2.481	0.009
Psychotic disorders	1.192	1.151	1.234	0.003
Dementia	1.193	1.152	1.234	<0.001

We divided the subjects with allergic diseases into six groups: bronchial asthma alone, allergic rhinitis alone, atopic dermatitis alone, bronchial asthma + allergic rhinitis, bronchial asthma + atopic dermatitis, allergic rhinitis + atopic dermatitis, and a combination of all these three allergic diseases. In this study, we found that the groups of atopic dermatitis alone and allergic rhinitis + atopic dermatitis were associated with a lower risk of psychiatric disorders, but the other four groups were associated with a higher risk of psychiatric disorders (Table 5).

**Table 5:** Factors of psychiatric disorders by using Cox regression and Fine and Gray’s competing risk model.

	Competing risk in the model			

Allergic diseases		95% CI	95% CI	*p*
Without	Reference			
Asthma	1.780	1.719	1.842	<0.001
Allergic rhinitis	1.156	1.117	1.197	<0.001
Atopic dermatitis	0.256	0.247	0.265	0.031
Asthma + allergic rhinitis	1.770	1.713	1.896	<0.001
Asthma + atopic dermatitis	1.723	1.673	1.852	<0.001
Allergic rhinitis + atopic dermatitis	0.554	0.213	0.966	0.018
Asthma + allergic rhinitis + atopic dermatitis	3.702	1.613	24.978	0.027

### Risk of Psychiatric Disorders Stratified by Covariates

We analyzed the data by the stratification of the factors such as sex, age, marital status, education levels, urbanization level, geographic areas of residence, seasons of medical visits, monthly insured premiums, and levels of care from medical services providers. We found that the subjects, who were either male or female, married or not married, and in different educational years, urbanization levels, residence areas, seasons of visits, insured premiums, and levels of care, were associated with an increased risk of psychiatric disorders. In the different age groups, subjects aged < 20 years were associated with a lower risk of psychiatric disorder, while subjects in other age groups (≥20 years) were associated with a higher risk (Table 6).

**Table 6:** Factors of psychiatric disorders stratified by variables listed in the table by using Fine and Gray’s competing risk model.

Allergic diseases (with vs. without)	Competing risk in the model			

Stratified	Adjusted HR	95% CI	95% CI	*p*
**Total**	1.659	1.602	1.717	<0.001
**Gender**
Male	1.705	1.646	1.765	<0.001
Female	1.604	1.549	1.660	<0.001
**Age group (years)**
<20	0.832	0.804	0.861	0.027
20–49	2.752	2.658	2.849	0.007
≥50	1.651	1.595	1.709	0.001
**Married**
Without	1.631	1.575	1.688	0.004
With	1.660	1.603	1.718	0.001
**Education (years)**
<12	3.140	3.032	3.250	<0.001
≥12	1.266	1.222	1.310	0.011
**Insured premium (New Taiwan Dollars)**
<18,000	1.651	1.595	1.709	0.007
18,000–34,999	2.543	2.455	2.632	<0.001
≥35,000	1.457	1.407	1.508	0.015
Season				
Spring	1.599	1.545	1.655	<0.001
Summer	1.677	1.620	1.736	<0.001
Autumn	1.645	1.589	1.703	0.002
Winter	1.708	1.649	1.768	<0.001
**Urbanization level**
1 (the highest)	1.326	1.280	1.372	<0.001
2	1.634	1.578	1.691	<0.001
3	1.757	1.697	1.818	0.001
4 (the lowest)	1.883	1.818	1.949	<0.001
**Level of care**
Hospital center	1.402	1.354	1.451	<0.001
Regional hospital	1.315	1.270	1.361	0.017
Local hospital	2.023	1.954	2.094	<0.001

*PYs, person-years; adjusted HR, adjusted hazard ratio: adjusted for the variables listed in Table 1; CI, confidence interval*.

### HR Analysis of Psychiatric Disorders in Patients With Different Medications for the Treatment of Asthma

The different medications of treatment durations more than 30 days for the treatment of bronchial asthma were grouped into four classes by the number of subjects who had used these medications: oral prednisolone, inhaled steroids, beta-agonist, aminophylline, leukotriene receptor antagonists, and anti-IgE antibody (omalizumab). Oral prednisolone usage was associated with a lower risk of developing psychiatric disorders in bronchial asthma patients. Besides, usage of beta-agonist, aminophylline, and leukotriene receptor antagonists was also associated with a lower risk of developing psychiatric disorders while adjusted with all the covariates and comorbidities, either with or without prednisolone usage (Table 7).

**Table 7:** HRs of patients with psychiatric disorders by medication use compared with patients with oral prednisolone use.

Medications use	Psychiatric disorders *n*(%)	Crude HR (95% CI)	Adjusted HR (95% CI)	Without prednisolone Adjusted HR (95% CI)	With prednisolone Adjusted HR (95% CI)
**Prednisolone**					
Yes (*n* = 20,202)	2,011 (9.95)	0.706 (0.465–0.801)**	0.713 (0.472–0.813)**	N/A	N/A
No (*n* = 20.203)	2,849 (14.10)	Reference	Reference		

**Inhaled steroids**					
Yes (*n* = 8,612)	988 (11.47)	0.942 (0.802–1.051)	0.970 (0.834–1.167)	0.982 (0.857–1.266)	0.912 (0.818–1.109)
No (*n* = 31,793)	3,872 (12.18)	Reference	Reference	Reference	Reference

**Beta-agonist**					
Yes (*n* = 21,720)	2,041 (9.40)	0.623 (0.511–0.729)***	0.684 (0.522–0.800)**	0.697 (0.529–0.824)*	0.662 (0.513–0.789)**
No (*n* = 18,685)	2,819 (15.09)	Reference	Reference	Reference	Reference

**Aminophylline**					
Yes (*n* = 21,898)	2,020 (9.22)	0.601 (0.499–0.714)***	0.654 (0.513–0.824)**	0.632 (0.492–0.810)**	0.678 (0.527–0.883)*
No (*n* = 18,507)	2,840 (15.35)	Reference	Reference	Reference	Reference

**Leukotriene receptor antagonists**					
Yes (*n* = 3,345)	291 (8.70)	0.706 (0.459–0.816)**	0.710 (0.469–0.826)**	0.709 (0.468–0.825)**	0.712 (0.472–0.829)**
No (*n* = 37,060)	4,569 (12.33)	Reference	Reference	Reference	Reference

**Anti-IgE antibody**					
Yes (*n* = 27)	1 (3.70)	0.308 (0.007–0.995)*	0.420 (0.010–1.097)	0.683 (0.145–2.894)	No patients
No (*n* = 40,378)	4,859 (12.03)	Reference	Reference	Reference	Reference

*Adjusted HR, adjusted hazard ratio: adjusted for the variables listed in Table 1; CI, confidence interval*.

*p < 0.05.

**p < 0.01.

***p < 0.001.

## Discussion

In this study, we examined the association between the overall allergic diseases and the risk of psychiatric disorders. After adjusting the covariates, the adjusted HR was 1.659 for the subjects (95% CI = 1.602–1.717, *p* < 0.001) when compared with the control group. In other words, the adult patients with allergic diseases had a 1.66-fold increased risk of developing psychiatric disorders. The Kaplan–Meier analysis revealed that the study subjects had a significantly higher 15-year psychiatric disorders-free survival rate than the controls.

Bronchial asthma and allergic rhinitis were associated with the risk of overall psychiatric disorders, but atopic dermatitis was associated with a lower risk of psychiatric disorders. Bronchial asthma was associated with the risk of individual psychiatric disorders, such as dementia, anxiety disorders, depressive disorders, eating disorders, bipolar disorders, sleep disorders, and psychotic disorders, but allergic rhinitis was associated with a lower risk of dementia and an increased risk of other psychiatric disorders. However, the increasing number of allergic diseases was associated with an increased risk of psychiatric disorders. In comparison to the previous studies for individual allergic diseases in several psychiatric disorders, such as anxiety disorders, depressive disorders, bipolar disorders, schizophrenia, and ADHD (4, 6–8, 10–15, 43), this is the first study on the topic of the association of allergic diseases and the broader spectrum of psychiatric disorders. Atopic dermatitis seems to be an exception in these allergic diseases, by being associated with a lower risk of psychiatric disorders, even though previous reports found that atopic dermatitis are associated with ADHD and autism spectrum disorders (52–55). Further study is needed to clarify the association between atopic dermatitis and the risk of psychiatric disorders.

The results of this study showed the association between overall allergic diseases and the risk of psychiatric disorders, which echoed the findings of other studies on the association between the overall allergic or atopic diseases and the risk of psychiatric disorders: for example, children with eczema, asthma, or hay fever had more emotional, conduct, and hyperactivity problems in a cross-sectional study in Denmark (56), and atopic diseases might be associated with an elevated risk of developing depression in a birth cohort study in Finland (57). Several studies have reported the associations between bronchial asthma or other atopic diseases and psychiatric disorders, such as affective disorders, anxiety disorders, schizophrenia, substance-related disorders, autism-spectrum disorders, or ADHDs (9, 58–62). Some researchers have also reported the associations between atopic dermatitis and attention-deficit/hyperactivity disorder, or speech disorders in childhood (63), and the association between allergic rhinitis and the risk of suicides and depression (64). Furthermore, parental exposure to occupational asthmagens might also be associated with children autism-spectrum disorders (65). Previous studies have also reported the associations between other immune or inflammation-related diseases, such as periodontitis (66), gluten-related illnesses (67), amyotrophiclateral sclerosis (68), psoriasis (69), food or other allergies (70), and idiopathic environmental intolerance (71) and the risk of psychiatric disorders. Conversely, psychiatric disorders might also affect the immune systems (72). In this study, the overall allergic diseases were associated with affective disorders, anxiety disorders, psychotic disorders, and dementia, respectively. The interplay between allergic diseases and psychiatric disorders, therefore, warrant further study.

The underlying mechanism of the association between allergic diseases and psychiatric disorders remains unclear. The “orchestration” of the pro-inflammatory cytokines plays an important role in the pathogenesis of allergy-related diseases, such as asthma (73). Reports have shown that cytokines such as interleukin (IL)-6, tumor necrosis factor-alpha (TNF-α), IL-10, and monocyte chemoattractant protein-1/CCL2 might be well associated with depressive, bipolar, or anxiety disorders (74, 75). Reports also show that dementia is related to peripheral pro-inflammatory factors released by periodontal inflammatory mediators such as C-reactive protein, IL-6, haptoglobin, TNF-α, and fibrinogen (76–78). Furthermore, some studies have also found that inflammation play an important role in eating disorders (79, 80) and sleep disorders (81–83).

Additionally, we have included several psychosocial factors in the analysis, such as marital status, educational level, monthly insured premiums, urbanization level, and residence. In comparison to the allergy-disease subjects who sought medical help in the local hospitals, study subjects who received medical help from medical centers or regional hospitals were associated with a lower risk of psychiatric disorders. These findings suggest that disadvantageous socioeconomic factors might well contribute to the risk of psychiatric disorders.

We found that the study subjects of those aged < 20 years had a lower risk in developing psychiatric disorders. We therefore hypothesize that the reason for a lower risk is that the maximal follow-up time is 15 years, which might not have, as yet, reached the age of onset of the most major psychiatric disorders for the patients of allergic diseases (84). Further study is needed to clarify the association between age and the risk of psychiatric disorders in the patients with allergic diseases.

Furthermore, we found that oral prednisolone usage was associated with a lower risk of developing psychiatric disorders in bronchial asthma patients. Moreover, the usage of beta-agonist, aminophylline, and leukotriene receptor antagonists was also associated with a lower risk of developing psychiatric disorders, with or without prednisolone usage. The mechanisms and effects of these medications on the risk of psychiatric disorders in bronchial asthma patients merit further study.

### Limitations

The present study has several limitations that warrant consideration. First, similar to previous studies using the NHIRD on allergic diseases as aforementioned, not all the data were recorded in the NHIRD, and we were unable to evaluate the severity, weakness severity, laboratory parameters, or lung function examinations in asthma patients. Second, other factors, such as genetic, psychosocial, and environmental factors, were not included in the dataset. Third, for patients who have to pay for their own for over-the-counter drugs for the allergic diseases, their self-medications would not have been included in the NHIRD. There is no study about the rates of self-medications for these allergic diseases. However, due to the high coverage of medical providers (97%) and beneficiaries (more than 99%) of the NHI system in Taiwan, most of the people would ask for help from the NHI-contracted hospital or clinics for their allergic diseases.

### Strength of This Study

One of the primary strengths of this study is the use of ICD-9 codes, and a number of studies have demonstrated the accuracy and validity of several diagnoses in the NHIRD, including DM (85, 86), cancer (87–89), myocardial infarction (85, 90, 91), and central nervous system diseases, such as Tourette syndrome (92), and stroke (85, 93–95), outcomes (89), mortality (85, 96), or comorbidity (89, 96). In a wide spectrum of conditions, some studies have also demonstrated the concordance between Taiwan’s National Health Survey and the NHIRD on various diagnoses (97), medication usage (97), and health system utilizations (97, 98). Correspondingly, the long-term observation period from 2000 to 2015 allowed for more credibility, when compared with other similar studies, to propose physical mechanisms and plausible hypotheses. Finally, and most importantly, we have tried to explain the mutual biological and psychological mechanism between the allergic diseases and the psychiatric disorders.

## Conclusion

We have evaluated the risk of psychiatric disorders in association with the allergic diseases such as asthma, allergic rhinitis, and atopic dermatitis in Taiwan’s population, using the representative population-based data. We have demonstrated that the patients with allergic diseases were at a significantly higher risk of psychiatric disorders than the control group. Further studies are therefore needed for patients with allergic diseases not only to prevent its clinical exacerbation but also to decrease the possibility of developing psychiatric disorders. We sincerely hope that this study will provide the necessary information for an earlier intervention for patients with allergic diseases.

## Ethics Statement

The Institutional Review Board of the Tri-Service General Hospital approved this study and waived the need for individual written informed consent (IRB No. 2-105-05-040 and IRB No. 2-105-05-082).

## Author Contributions

N-ST, H-AC, and W-CC conceived, planned, and conducted this study. C-HC and W-SC contributed to the data analysis and interpretation. Y-CK, C-CC, H-WY, Y-CC, and S-Y contributed to this data interpretation. N-ST wrote the draft. All the authors approved this manuscript.

## Conflict of Interest Statement

The authors declare that the research was conducted in the absence of any commercial or financial relationships that could be construed as a potential conflict of interest.

## References

[1] Hong S Son DK Lim WR Kim SH Kim H Yum HY The prevalence of atopic dermatitis, asthma, and allergic rhinitis and the comorbidity of allergic diseases in children. Environ Health Toxicol (2012) 27:e2012006. 10.5620/eht.2012.27.e2012006 22359737 PMC3282234

[2] Grize L Gassner M Wuthrich B Bringolf-Isler B Takken-Sahli K Sennhauser FH Trends in prevalence of asthma, allergic rhinitis and atopic dermatitis in 5–7-year old Swiss children from 1992 to 2001. Allergy (2006) 61(5):556–62. 10.1111/j.1398-9995.2006.01030.x 16629784

[3] Yan DC Ou LS Tsai TL Wu WF Huang JL. Prevalence and severity of symptoms of asthma, rhinitis, and eczema in 13- to 14-year-old children in Taipei, Taiwan. Ann Allergy Asthma Immunol (2005) 95(6):579–85. 10.1016/S1081-1206(10)61022-8 16400899

[4] Lee YC Lee CT Lai YR Chen VC Stewart R. Association of asthma and anxiety: a nationwide population-based study in Taiwan. J Affect Disord (2016) 189:98–105. 10.1016/j.jad.2015.09.040 26432033

[5] Lin TC Lee CT Lai TJ Lee CT Lee KY Chen VC Association of asthma and bipolar disorder: a nationwide population-based study in Taiwan. J Affect Disord (2014) 168:30–6. 10.1016/j.jad.2014.06.033 25033475

[6] Chen MH Su TP Chen YS Hsu JW Huang KL Chang WH Higher risk of developing major depression and bipolar disorder in later life among adolescents with asthma: a nationwide prospective study. J Psychiatr Res (2014) 49:25–30. 10.1016/j.jpsychires.2013.10.015 24275549

[7] Chen MH Su TP Chen YS Hsu JW Huang KL Chang WH Asthma and attention-deficit/hyperactivity disorder: a nationwide population-based prospective cohort study. J Child Psychol Psychiatry (2013) 54(11):1208–14. 10.1111/jcpp.12087 23730913

[8] Chen MH Su TP Chen YS Hsu JW Huang KL Chang WH Attention deficit hyperactivity disorder, tic disorder, and allergy: is there a link? A nationwide population-based study. J Child Psychol Psychiatry (2013) 54(5):545–51. 10.1111/jcpp.12018 23140273

[9] Wang WC Lu ML Chen VC Ng MH Huang KY Hsieh MH Asthma, corticosteroid use and schizophrenia: a nationwide population-based study in Taiwan. PLoS One (2017) 12(3):e0173063. 10.1371/journal.pone.0173063 28350822 PMC5369699

[10] Chen MH Li CT Tsai CF Lin WC Chang WH Chen TJ Risk of dementia among patients with asthma: a nationwide longitudinal study. J Am Med Dir Assoc (2014) 15(10):763–7. 10.1016/j.jamda.2014.06.003 25037169

[11] Chen MH Su TP Chen YS Hsu JW Huang KL Chang WH Allergic rhinitis in adolescence increases the risk of depression in later life: a nationwide population-based prospective cohort study. J Affect Disord (2013) 145(1):49–53. 10.1016/j.jad.2012.07.011 22889525

[12] Hsu CL Wang TC Shen TC Huang YJ Lin CL Sung FC. Risk of depression in patients with chronic rhinosinusitis: a nationwide population-based retrospective cohort study. J Affect Disord (2016) 206:294–9. 10.1016/j.jad.2016.09.001 27643962

[13] Chen MH Lan WH Hsu JW Huang KL Chen YS Li CT Risk of bipolar disorder among adolescents with allergic rhinitis: a nationwide longitudinal study. J Psychosom Res (2015) 79(6):533–6. 10.1016/j.jpsychores.2015.08.009 26363680

[14] Cheng CM Hsu JW Huang KL Bai YM Su TP Li CT Risk of developing major depressive disorder and anxiety disorders among adolescents and adults with atopic dermatitis: a nationwide longitudinal study. J Affect Disord (2015) 178:60–5. 10.1016/j.jad.2015.02.025 25795537

[15] Chen YH Lee HC Lin HC. Prevalence and risk of atopic disorders among schizophrenia patients: a nationwide population based study. Schizophr Res (2009) 108(1–3):191–6. 10.1016/j.schres.2008.12.021 19171465

[16] American Psychiatric Association. Diagnostic and Statistical Manual of Mental Disorders. 4th ed USA: American Psychiatric Association (1994).

[17] Stein DJ Phillips KA Bolton D Fulford KW Sadler JZ Kendler KS. What is a mental/psychiatric disorder? From DSM-IV to DSM-V. Psychol Med (2010) 40(11):1759–65. 10.1017/S0033291709992261 20624327 PMC3101504

[18] Marrie RA Walld R Bolton JM Sareen J Walker JR Patten SB Rising incidence of psychiatric disorders before diagnosis of immune-mediated inflammatory disease. Epidemiol Psychiatr Sci (2017):1–10. 10.1017/S2045796017000579 29098977 PMC6998907

[19] Marrie RA Walld R Bolton JM Sareen J Walker JR Patten SB Increased incidence of psychiatric disorders in immune-mediated inflammatory disease. J Psychosom Res (2017) 101:17–23. 10.1016/j.jpsychores.2017.07.015 28867419

[20] Marrie RA Reingold S Cohen J Stuve O Trojano M Sorensen PS The incidence and prevalence of psychiatric disorders in multiple sclerosis: a systematic review. Mult Scler (2015) 21(3):305–17. 10.1177/1352458514564490 25583845 PMC4429164

[21] Marrie RA Horwitz R Cutter G Tyry T Campagnolo D Vollmer T. The burden of mental comorbidity in multiple sclerosis: frequent, underdiagnosed, and undertreated. Mult Scler (2009) 15(3):385–92. 10.1177/1352458508099477 19153176

[22] Chao PC Chien WC Chung CH Chu CW Yeh CB Huang SY Cognitive enhancers associated with decreased risk of injury in patients with dementia: a nationwide cohort study in Taiwan. J Investig Med (2017) 66(3):684–92. 10.1136/jim-2017-000595 29141875

[23] Cunningham NR Tran ST Lynch-Jordan AM Ting TV Sil S Strotman D Psychiatric disorders in young adults diagnosed with juvenile fibromyalgia in Adolescence. J Rheumatol (2015) 42(12):2427–33. 10.3899/jrheum.141369 26373565 PMC4668223

[24] Chen YT Su JS Tseng CW Chen CC Lin CL Kao CH. Inflammatory bowel disease on the risk of acute pancreatitis: a population-based cohort study. J Gastroenterol Hepatol (2016) 31(4):782–7. 10.1111/jgh.13171 26412125

[25] Suarez AL Feramisco JD Koo J Steinhoff M. Psychoneuroimmunology of psychological stress and atopic dermatitis: pathophysiologic and therapeutic updates. Acta Derm Venereol (2012) 92(1):7–15. 10.2340/00015555-1188 22101513 PMC3704139

[26] Sgambato D Miranda A Ranaldo R Federico A Romano M The role of stress in inflammatory bowel diseases. Curr Pharm Des (2017) 23(27):3997–4002. 10.2174/1381612823666170228123357 28245757

[27] Voorhees JL Tarr AJ Wohleb ES Godbout JP Mo X Sheridan JF Prolonged restraint stress increases IL-6, reduces IL-10, and causes persistent depressive-like behavior that is reversed by recombinant IL-10. PLoS One (2013) 8(3):e58488. 10.1371/journal.pone.0058488 23520517 PMC3592793

[28] Trueba AF Ritz T Trueba G. The role of the microbiome in the relationship of asthma and affective disorders. Adv Exp Med Biol (2016) 874:263–88. 10.1007/978-3-319-20215-0_13 26589224

[29] Altamura AC Buoli M Pozzoli S. Role of immunological factors in the pathophysiology and diagnosis of bipolar disorder: comparison with schizophrenia. Psychiatry Clin Neurosci (2014) 68(1):21–36. 10.1111/pcn.12089 24102953

[30] Ho Chan W. Taiwan’s healthcare report 2010. EPMA J (2010) 1(4):563–85. 10.1007/s13167-010-0056-8 23199110 PMC3405348

[31] Needham DM Scales DC Laupacis A Pronovost PJ. A systematic review of the Charlson comorbidity index using Canadian administrative databases: a perspective on risk adjustment in critical care research. J Crit Care (2005) 20(1):12–9. 10.1016/j.jcrc.2004.09.007 16015512

[32] Chinese Hospital Association. ICD-9-CM English-Chinese Dictionary. Taipei, Taiwan: Chinese Hospital Association Press (2000).

[33] Huang HL Ho SY Li CH Chu FY Ciou LP Lee HC Bronchial asthma is associated with increased risk of chronic kidney disease. BMC Pulm Med (2014) 14:80. 10.1186/1471-2466-14-80 24885269 PMC4022436

[34] Tzeng NS Hsu YH Ho SY Kuo YC Lee HC Yin YJ Is schizophrenia associated with an increased risk of chronic kidney disease? A nationwide matched-cohort study. BMJ Open (2015) 5(1):e006777. 10.1136/bmjopen-2014-006777 25628048 PMC4316552

[35] Tang YJ Ho SY Chu FY Chen HA Yin YJ Lee HC Is zolpidem associated with increased risk of fractures in the elderly with sleep disorders? A nationwide case cross-over study in Taiwan. PLoS One (2015) 10(12):e0146030. 10.1371/journal.pone.0146030 26716836 PMC4700989

[36] Yang CW Tzeng NS Yin YJ Li CH Chen HA Chiu SH Angiotensin receptor blockers decrease the risk of major adverse cardiovascular events in patients with end-stage renal disease on maintenance dialysis: a nationwide matched-cohort study. PLoS One (2015) 10(10):e0140633. 10.1371/journal.pone.0140633 26488749 PMC4619342

[37] Tzeng NS Chung CH Yeh CB Huang RY Yuh DY Huang SY Are chronic periodontitis and gingivitis associated with dementia? A nationwide, retrospective, matched-cohort study in Taiwan. Neuroepidemiology (2016) 47(2):82–93. 10.1159/000449166 27618156

[38] Tzeng NS Chang HA Chung CH Lin FH Yeh CB Huang SY Risk of psychiatric disorders in Guillain-Barre syndrome: a nationwide, population-based, cohort study. J Neurol Sci (2017) 381:88–94. 10.1016/j.jns.2017.08.022 28991722

[39] Tzeng NS Chung CH Lin FH Huang CF Yeh CB Huang SY Magnesium oxide use and reduced risk of dementia: a retrospective, nationwide cohort study in Taiwan. Curr Med Res Opin (2017) 34(1):163–9. 10.1080/03007995.2017.1385449 28952385

[40] Tzeng NS Chung CH Lin FH Yeh CB Huang SY Lu RB Headaches and risk of dementia. Am J Med Sci (2017) 353(3):197–206. 10.1016/j.amjms.2016.12.014 28262204

[41] Chien WC Chung CH Lin FH Chang HA Kao YC Tzeng NS Is weight control surgery associated with increased risk of newly onset psychiatric disorders? A population-based, matched cohort study in Taiwan. J Med Sci (2017) 37(4):137–49. 10.4103/jmedsci.jmedsci_94_16

[42] Tzeng NS Chung CH Lin FH Yeh CB Huang SY Lu RB Risk of dementia in adults with ADHD: a nationwide, population-based cohort study in Taiwan. J Atten Disord (2017). 10.1177/1087054717714057 28629260

[43] Chang CY Chen WL Liou YF Ke CC Lee HC Huang HL Increased risk of major depression in the three years following a femoral neck fracture – a national population-based follow-up study. PLoS One (2014) 9(3):e89867 10.1371/journal.pone.0089867 24626193 PMC3953077

[44] McGrogan A Madle GC Seaman HE de Vries CS The epidemiology of Guillain-Barré syndrome worldwide. Neuroepidemiology (2009) 32(2):150–63. 10.1159/000184748 19088488

[45] van den Berg B Walgaard C Drenthen J Fokke C Jacobs BC van Doorn PA Guillain-Barre syndrome: pathogenesis, diagnosis, treatment and prognosis. Nat Rev Neurol (2014) 10(8):469–82. 10.1038/nrneurol.2014.121 25023340

[46] Willison HJ Jacobs BC van Doorn PA Guillain-Barre syndrome: surveillance and cost of treatment strategies – authors’ reply. Lancet (2017) 389(10066):253–4. 10.1016/S0140-6736(17)30047-8 28118915

[47] Group SG-BST. Randomised trial of plasma exchange, intravenous immuno-globulin, and combined treatments in Guillain-Barre syndrome. Plasma exchange. Lancet (1997) 349(9047):225–30. 10.1016/S0140-6736(96)09095-2 9014908

[48] van den Berg B Bunschoten C van Doorn PA Jacobs BC. Mortality in Guillain-Barre syndrome. Neurology (2013) 80(18):1650–4. 10.1212/WNL.0b013e3182904fcc 23576619

[49] Wong AHY Umapathi T Shahrizaila N Chan YC Kokubun N Fong MK The value of comparing mortality of Guillain-Barré syndrome across different regions. J Neurol Sci (2014) 344(1–2):60–2. 10.1016/j.jns.2014.06.021 24993467

[50] Marzona I Baviera M Vannini T Tettamanti M Cortesi L Riva E Risk of dementia and death in patients with atrial fibrillation: a competing risk analysis of a population-based cohort. Int J Cardiol (2016) 220:440–4. 10.1016/j.ijcard.2016.06.235 27394970

[51] Blanche P Proust-Lima C Loubere L Berr C Dartigues JF Jacqmin-Gadda H. Quantifying and comparing dynamic predictive accuracy of joint models for longitudinal marker and time-to-event in presence of censoring and competing risks. Biometrics (2015) 71(1):102–13. 10.1111/biom.12232 25311240

[52] Billeci L Tonacci A Tartarisco G Ruta L Pioggia G Gangemi S. Association between atopic dermatitis and autism spectrum disorders: a systematic review. Am J Clin Dermatol (2015) 16(5):371–88. 10.1007/s40257-015-0145-5 26254000

[53] Lee CY Chen MH Jeng MJ Hsu JW Tsai SJ Bai YM Longitudinal association between early atopic dermatitis and subsequent attention-deficit or autistic disorder: a population-based case-control study. Medicine (2016) 95(39):e5005. 10.1097/MD.0000000000005005 27684861 PMC5265954

[54] Liao TC Lien YT Wang S Huang SL Chen CY. Comorbidity of atopic disorders with autism spectrum disorder and attention deficit/hyperactivity disorder. J Pediatr (2016) 171:248–55. 10.1016/j.jpeds.2015.12.063 26846570

[55] Strom MA Fishbein AB Paller AS Silverberg JI. Association between atopic dermatitis and attention deficit hyperactivity disorder in U.S. children and adults. Br J Dermatol (2016) 175(5):920–9. 10.1111/bjd.14697 27105659 PMC5216180

[56] Hammer-Helmich L Linneberg A Obel C Thomsen SF Tang Mollehave L Glumer C. Mental health associations with eczema, asthma and hay fever in children: a cross-sectional survey. BMJ Open (2016) 6(10):e012637. 10.1136/bmjopen-2016-012637 27742629 PMC5073477

[57] Timonen M Jokelainen J Hakko H Silvennoinen-Kassinen S Meyer-Rochow VB Herva A Atopy and depression: results from the Northern Finland 1966 Birth Cohort Study. Mol Psychiatry (2003) 8(8):738–44. 10.1038/sj.mp.4001274 12888802

[58] Jonsdottir U Lang JE. How does autism spectrum disorder affect the risk and severity of childhood asthma? Ann Allergy Asthma Immunol (2017) 118(5):570–6. 10.1016/j.anai.2017.02.020 28477788

[59] Heck S Al-Shobash S Rapp D Le DD Omlor A Bekhit A High probability of comorbidities in bronchial asthma in Germany. NPJ Prim Care Respir Med (2017) 27(1):28. 10.1038/s41533-017-0026-x 28432297 PMC5435094

[60] To T Ryckman K Zhu J Williams D Feldman LY Larsen K Mental health services claims and adult onset asthma in Ontario, Canada. J Allergy Clin Immunol Pract (2017) 5(5):1388–93.e3. 10.1016/j.jaip.2017.02.016 28396111

[61] Miyazaki C Koyama M Ota E Swa T Mlunde LB Amiya RM Allergic diseases in children with attention deficit hyperactivity disorder: a systematic review and meta-analysis. BMC Psychiatry (2017) 17(1):120. 10.1186/s12888-017-1281-7 28359274 PMC5374627

[62] Schans JV Cicek R de Vries TW Hak E Hoekstra PJ. Association of atopic diseases and attention-deficit/hyperactivity disorder: a systematic review and meta-analyses. Neurosci Biobehav Rev (2017) 74(Pt A):139–48. 10.1016/j.neubiorev.2017.01.011 28111269

[63] Silverberg JI. Selected comorbidities of atopic dermatitis: atopy, neuropsychiatric, and musculoskeletal disorders. Clin Dermatol (2017) 35(4):360–6. 10.1016/j.clindermatol.2017.03.008 28709566 PMC5512438

[64] Amritwar AU Lowry CA Brenner LA Hoisington AJ Hamilton R Stiller JW Mental health in allergic rhinitis: depression and suicidal behavior. Curr Treat Options Allergy (2017) 4(1):71–97. 10.1007/s40521-017-0110-z 28966902 PMC5614510

[65] Singer AB Burstyn I Thygesen M Mortensen PB Fallin MD Schendel DE. Parental exposures to occupational asthmagens and risk of autism spectrum disorder in a Danish population-based case-control study. Environ Health (2017) 16(1):31. 10.1186/s12940-017-0230-8 28359263 PMC5374665

[66] Sperr M Kundi M Tursic V Bristela M Moritz A Andrukhov O Prevalence of comorbidities in periodontitis patients compared to the general Austrian population. J Periodontol (2017) 89:1–13. 10.1902/jop.2017.170333 28844189

[67] Brietzke E Cerqueira RO Mansur RB McIntyre RS. Gluten related illnesses and severe mental disorders: a comprehensive review. Neurosci Biobehav Rev (2018) 84:368–75. 10.1016/j.neubiorev.2017.08.009 28830676

[68] Longinetti E Mariosa D Larsson H Ye W Ingre C Almqvist C Neurodegenerative and psychiatric diseases among families with amyotrophic lateral sclerosis. Neurology (2017) 89(6):578–85. 10.1212/WNL.0000000000004179 28701495 PMC5562958

[69] Pompili M Innamorati M Forte A Erbuto D Lamis DA Narcisi A Psychiatric comorbidity and suicidal ideation in psoriasis, melanoma and allergic disorders. Int J Psychiatry Clin Pract (2017) 21(3):209–14. 10.1080/13651501.2017.1301482 28326880

[70] Aberle D Wu SE Oklu R Erinjeri J Deipolyi AR. Association between allergies and psychiatric disorders in patients undergoing invasive procedures. Psychosomatics (2017) 58(5):490–5. 10.1016/j.psym.2017.03.015 28527521

[71] Weiss EM Singewald E Baldus C Hofer E Marksteiner J Nasrouei S Differences in psychological and somatic symptom cluster score profiles between subjects with idiopathic environmental intolerance, major depression and schizophrenia. Psychiatry Res (2017) 249:187–94. 10.1016/j.psychres.2016.12.057 28113122

[72] Larsen JB Iversen VC Reitan SK Association of psychosis, affective disorders and diseases affecting the immune system. Nord J Psychiatry (2017) 72:145–9. 10.1080/08039488.2017.1402952 29141491

[73] Castillo EF Zheng H Yang XO Orchestration of epithelial-derived cytokines and innate immune cells in allergic airway inflammation. Cytokine Growth Factor Rev (2017) 39:19–25. 10.1016/j.cytogfr.2017.11.004 29169815 PMC5866749

[74] Kohler CA Freitas TH Stubbs B Maes M Solmi M Veronese N Peripheral alterations in cytokine and chemokine levels after antidepressant drug treatment for major depressive disorder: systematic review and meta-analysis. Mol Neurobiol (2017). 10.1007/s12035-017-0632-1 28612257

[75] Rosenblat JD McIntyre RS. Bipolar disorder and immune dysfunction: epidemiological findings, proposed pathophysiology and clinical implications. Brain Sci (2017) 7(11):E144. 10.3390/brainsci7110144 29084144 PMC5704151

[76] D’Aiuto F Graziani F Tete S Gabriele M Tonetti MS. Periodontitis: from local infection to systemic diseases. Int J Immunopathol Pharmacol (2005) 18(3 Suppl):1–11. 16848982

[77] Farhad SZ Amini S Khalilian A Barekatain M Mafi M Barekatain M The effect of chronic periodontitis on serum levels of tumor necrosis factor-alpha in Alzheimer disease. Dent Res J (Isfahan) (2014) 11(5):549–52. 25426144 PMC4241606

[78] Holmes C El-Okl M Williams AL Cunningham C Wilcockson D Perry VH. Systemic infection, interleukin 1beta, and cognitive decline in Alzheimer’s disease. J Neurol Neurosurg Psychiatry (2003) 74(6):788–9. 10.1136/jnnp.74.6.788 12754353 PMC1738504

[79] Raevuori A Lukkariniemi L Suokas JT Gissler M Suvisaari JM Haukka J. Increased use of antimicrobial medication in bulimia nervosa and binge-eating disorder prior to the eating disorder treatment. Int J Eat Disord (2016) 49(6):542–52. 10.1002/eat.22497 26875554

[80] Succurro E Segura-Garcia C Ruffo M Caroleo M Rania M Aloi M Obese patients with a binge eating disorder have an unfavorable metabolic and inflammatory profile. Medicine (2015) 94(52):e2098. 10.1097/MD.0000000000002098 26717356 PMC5291597

[81] Troester N Palfner M Schmidberger E Olschewski H Avian A Sleep related breathing disorders and inflammation – the missing link? A Cohort study evaluating the interaction of inflammation and sleep related breathing disorders and effects of treatment. PLoS One (2015) 10(9):e0137594 10.1371/journal.pone.0137594 26356577 PMC4565554

[82] Irwin MR Olmstead R Breen EC Witarama T Carrillo C Sadeghi N Cognitive behavioral therapy and tai chi reverse cellular and genomic markers of inflammation in late-life insomnia: a randomized controlled trial. Biol Psychiatry (2015) 78(10):721–9. 10.1016/j.biopsych.2015.01.010 25748580 PMC4524803

[83] Irwin MR Opp MR. Sleep health: reciprocal regulation of sleep and innate immunity. Neuropsychopharmacology (2017) 42(1):129–55. 10.1038/npp.2016.148 27510422 PMC5143488

[84] Yin H Xu G Tian H Yang G Wardenaar KJ Schoevers RA The prevalence, age-of-onset and the correlates of DSM-IV psychiatric disorders in the Tianjin Mental Health Survey (TJMHS). Psychol Med (2017) 48(3):473–87. 10.1017/S0033291717001878 28714421

[85] Cheng CL Chien HC Lee CH Lin SJ Yang YH. Validity of in-hospital mortality data among patients with acute myocardial infarction or stroke in national health insurance research database in Taiwan. Int J Cardiol (2015) 201:96–101. 10.1016/j.ijcard.2015.07.075 26292275

[86] Lin CC Lai MS Syu CY Chang SC Tseng FY. Accuracy of diabetes diagnosis in health insurance claims data in Taiwan. J Formos Med Assoc (2005) 104(3):157–63. 15818428

[87] Liang JA Sun LM Muo CH Sung FC Chang SN Kao CH. The analysis of depression and subsequent cancer risk in Taiwan. Cancer Epidemiol Biomarkers Prev (2011) 20(3):473–5. 10.1158/1055-9965.EPI-10-1280 21297039

[88] Li-Ting C Chung-Ho C Yi-Hsin Y Pei-Shan H. The development and validation of oral cancer staging using administrative health data. BMC Cancer (2014) 14:380. 10.1186/1471-2407-14-380 24884513 PMC4049423

[89] Yang CC Chen PC Hsu CW Chang SL Lee CC. Validity of the age-adjusted charlson comorbidity index on clinical outcomes for patients with nasopharyngeal cancer post radiation treatment: a 5-year nationwide cohort study. PLoS One (2015) 10(1):e0117323. 10.1371/journal.pone.0117323 25617629 PMC4305297

[90] Cheng CL Lee CH Chen PS Li YH Lin SJ Yang YH. Validation of acute myocardial infarction cases in the national health insurance research database in taiwan. J Epidemiol (2014) 24(6):500–7. 10.2188/jea.JE20140076 25174915 PMC4213225

[91] Hsieh CY Chen CH Li CY Lai ML. Validating the diagnosis of acute ischemic stroke in a national health insurance claims database. J Formos Med Assoc (2015) 114(3):254–9. 10.1016/j.jfma.2013.09.009 24140108

[92] Chou IC Lin HC Lin CC Sung FC Kao CH. Tourette syndrome and risk of depression: a population-based cohort study in Taiwan. J Dev Behav Pediatr (2013) 34(3):181–5. 10.1097/DBP.0b013e3182829f2b 23572168

[93] Cheng CL Kao YH Lin SJ Lee CH Lai ML. Validation of the national health insurance research database with ischemic stroke cases in Taiwan. Pharmacoepidemiol Drug Saf (2011) 20(3):236–42. 10.1002/pds.2087 21351304

[94] Sung SF Hsieh CY Lin HJ Chen YW Yang YH Li CY. Validation of algorithms to identify stroke risk factors in patients with acute ischemic stroke, transient ischemic attack, or intracerebral hemorrhage in an administrative claims database. Int J Cardiol (2016) 215:277–82. 10.1016/j.ijcard.2016.04.069 27128546

[95] Tseng HP Lin FJ Chen PT Mou CH Lee SP Chang CY Derivation and validation of a discharge disposition predicting model after acute stroke. J Stroke Cerebrovasc Dis (2015) 24(6):1179–86. 10.1016/j.jstrokecerebrovasdis.2015.01.010 25847306

[96] Yang H Chen YH Hsieh TF Chuang SY Wu MJ. Prediction of mortality in incident hemodialysis patients: a validation and comparison of CHADS2, CHA2DS2, and CCI scores. PLoS One (2016) 11(5):e0154627. 10.1371/journal.pone.0154627 27148867 PMC4858249

[97] Wu CS Lai MS Gau SS Wang SC Tsai HJ. Concordance between patient self-reports and claims data on clinical diagnoses, medication use, and health system utilization in Taiwan. PLoS One (2014) 9(12):e112257. 10.1371/journal.pone.0112257 25464005 PMC4251897

[98] Yu ST Chang HY Lin MC Lin YH. Agreement between self-reported and health insurance claims on utilization of health care: a population study. J Clin Epidemiol (2009) 62(12):1316–22. 10.1016/j.jclinepi.2009.01.016 19403264

